# The complete mitochondrial genome of *Hericium coralloides* (Hericiaceae, Basidiomycota)

**DOI:** 10.1080/23802359.2017.1347898

**Published:** 2017-07-07

**Authors:** Caixia Zhang, Changshan Li, Weixing Ye, Manjun Yang

**Affiliations:** a Tibet Vocational Technical College, Lhasa, Xizang, People’s Republic of China;; b Shanghai Personal Biotechnology Co., Ltd, Shanghai, People’s Republic of China;; c School of Life Sciences, Sun Yatsen University, University City, Guangzhou, People’s Republic of China

**Keywords:** *Hericium coralloides*, mitochondrial genome, coral-like Monkey Head mushroom

## Abstract

In this study, the complete mitochondrial genome of the medicinal mushroom *Hericium coralloides* (Hericiaceae, Basidiomycota) was sequenced. This mitochondrial genome is 72,961 bp in length and consisted of 14 protein-coding genes, 21 hypothetical open reading frames, 2 ribosomal RNA subunits and 27 transfer RNAs. The overall nucleotide composition of is 41.33% A, 40.71% T, 9.06% C and 8.90% G, with GC content of 17.96%. A phylogenetic tree with the complete mitochondrial genome sequences of *Hericium coralloides* together with 9 other affinis mushrooms was constructed. The newly achieved mitochondrial genome sequence seem to be useful for addressing taxonomic issues and studying related evolution events, which would contribute to enrich the fungal mitochondrial genome resource and promote the biological research.

The edible and medical fungus, *Hericium coralloides* (Hericiaceae, Basidiomycota) is a species of the genus *Hericium* with specific features. For example, *Hericium coralloides* is a white fleshy and coral-like fungus growing mostly on dead or rotting hardwood (McCracken and Dodd, 1971). The fruiting bodies of *H. coralloides* are edible and considered as a delicious mushroom, and it has long been used in traditional Chinese medicine (TCM) to treat diseases (Wittstein et al. 2016). In this study, we present the complete mitochondrial genome of *Hericium coralloides* (GenBank accession no: KY007042, NCBI Reference Sequence: NC_033903.1).

The complete sequence of mitochondrial DNA of *H. coralloides* is 72,961 bp in length. The overall nucleotide composition of is 41.33% A, 40.71% T, 9.06% C and 8.90% G, with GC content of 17.96%. The gene order and content of *H. coralloides* consisted of the large and small rRNA genes (*rnl* and *rns*), 27 tRNAs, 21 hypothetical open reading frames (ORFs), and a core set of 14 protein-coding genes (PCGs) of the seven subunits of NAD dehydrogenase (*nad1-6* and *nad4L* genes), three cytochrome oxidases (*cox1-3*), apocytochrome b (*cob*), three ATP synthases (*atp6, 8, 9*). The tRNA genes contain codons for all 20 standard amino acids and an additional other codon for *trnX-AGCC*. Most amino acids are represented by only one tRNA gene; however, two *trnR* (*trnR-UCG* and *trnR-UCU*), two *trnL*(*trnL-UAA* and *trnL-UAG*), two *trnS* (*trnS-GCU* and *trnS-UGA*), two *trnW* (*trnW-CCA* and *trnW-UCA*), and three *trnM-CAU* were identified in this mitochondrial genome.

Compared to mitochondrial genome of *Heterobasidion irregular* (114.19 kb), the genome size of *H. coralloides* (72.96 kb) is much smaller (Himmelstrand et al. 2014). *rps3*, a core set of protein-coding genes, is absent from the genome of *H. coralloides*, and none of genes in *H. coralloides* contains introns. All PCGs and RNA genes are encoded on the H-strand except for 2 tRNAs genes and 1 PCGs in *H. irregular*, while they seem to be evenly encoded on both H- and L-strand in *H. coralloides*.

In order to verify the evolutionary relationship, we constructed a phylogenetic tree with the complete mitochondrial genome sequences of *H. coralloides* together with nine other affinis mushrooms from NCBI, including *Agaricus bisporus* var. bisporus H97, *Lentinula edodes*, *Tricholoma matsutake*, *Ganoderma lucidum, Pleurotus ostreatus*, *Pleurotus eryngii*, *Cordyceps militaris* isolate CM09-31-28, *Flammulina velutipes* strain 4019-20, *Annulohypoxylon stygium*. The phylogenetic tree was constructed using Clustal W 2.1 (Larkin et al. 2007) and TreeView 1.6.6 (Page RD, 1996) based on the neighbour-joining (NJ) method (Saitou & Nei 1987). The topology clearly illustrated phylogenetic relationship of *H. coralloides* and these related species (Figure 1). The phylogenetic data are consistent with the previous report (Li et al. 2013), indicating that our newly achieved mitochondrial genome sequence could meet the quality demands and provide insights into certain evolution issues.

**Figure 1.**
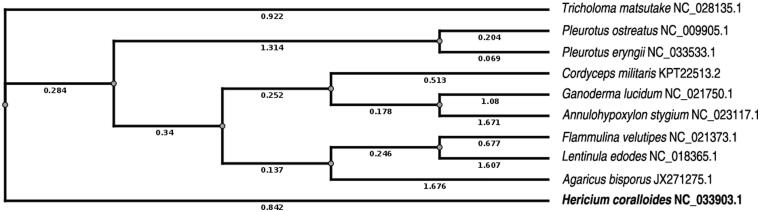
Neighbor-joining phylogenetic tree based on the mitochondrial genome sequences of *H. coralloides* and other 9 affinis mushrooms using Clustal W and TreeView. The genbank accession numbers of 10 related species are as follows: *Tricholoma matsutake*, NC_028135.1; *Pleurotus ostreatus*, NC_009905.1; *Pleurotus eryngii*, NC_033533.1; *Cordyceps militaris*, KPT22513.2; *Ganoderma lucidum*, NC_021750.1; *Annulohypoxylon stygium*, NC_023117.1; *Flammulina velutipes*, NC_021373.1; Lentiula edodes, NC_018365.1; *Agaricus bisporus*, JX271275.1; *Hericium coralloides*, NC_033903.1.
